# Dihydroartemisinin-piperaquine effectiveness for seasonal malaria chemoprevention in settings with extended seasonal malaria transmission in Tanzania

**DOI:** 10.1038/s41598-024-52706-z

**Published:** 2024-01-25

**Authors:** Richard Mwaiswelo, Billy Ngasala, Frank Chaky, Fabrizio Molteni, Ally Mohamed, Samwel Lazaro, Bushukatale Samwel, Bruno P. Mmbando

**Affiliations:** 1 Department of Microbiology, Immunology, and Parasitology, Faculty of Medicine, Hubert Kairuki Memorial University, Dar es Salaam, Tanzania; 2 Department of Medical Parasitology and Entomology, Muhimbili University of Health and Allied Sciences, Dar es Salaam, Tanzania; 3 National Malaria Control Programme, Ministry of Health, Dodoma, Tanzania; 4 Swiss Tropical and Public Health Institute, Basel, Switzerland; 5 National Institute for Medical Research, Tanga Research Centre, Tanga, Tanzania

**Keywords:** Health care, Medical research

## Abstract

Effectiveness of dihydroartemisinin-piperaquine (DP) as seasonal malaria chemoprevention (SMC) was assessed in Nanyumbu and Masasi Districts. Between March and June 2021, children aged 3–59 months were enrolled in a cluster randomized study. Children in the intervention clusters received a monthly, 3-days course of DP for three consecutive months regardless of malaria infection status, and those in the control clusters received no intervention. Malaria infection was assessed at before the first-round and at 7 weeks after the third-round of DP in both arms. Malaria prevalence after the third-round of DP administration was the primary outcome. *Chi*-square tests and logistic regression model were used to compare proportions and adjust for explanatory variables. Before the intervention, malaria prevalence was 13.7% (161/1171) and 18.2% (212/1169) in the intervention and control clusters, respectively, *p* < 004. Malaria prevalence declined to 5.8% (60/1036) in the intervention clusters after three rounds of DP, and in the control clusters it declined to 9.3% (97/1048), *p* = 0.003. Unadjusted and adjusted prevalence ratios between the intervention and control arms were 0.42 (95%CI 0.32–0.55, *p* < 0.001) and 0.77 (95%CI 0.53–1.13, *p* = 0.189), respectively. SMC using DP was effective for control of malaria in the two Districts.

*Trial registration*: NCT05874869, https://clinicaltrials.gov/ 25/05/2023.

## Introduction

In the malaria-endemic settings of sub-Saharan Africa underfive children are the most affected by the infection^1–4^. Most of the malaria-related morbidity and mortality occur during the rainy season^5,6^. Administration of antimalarial chemoprophylaxis to the underfives at appropriate intervals during the rainy season (seasonal malaria chemoprevention (SMC) preferably at the start of the malaria transmission season, up to a maximum of four doses reduces malaria-related morbidity and mortality in the group^6^. The strategy is effective especially in areas where malaria transmission is highly seasonal^7,8^. SMC is recommended in areas where more than 60% of the annual rainfall and 60% of clinical malaria cases occur within 3 and 4 consecutive months^6,8^. In 2012 the World Health Organization (WHO) recommended SMC using sulphadoxine-pyrimethamine (SP) plus amodiaquine (AQ) to children aged 3–59 months^6,9–11^. Since then SMC using SP-AQ has been scaled-up in the Western sub-Sahel region countries^12^, and it has led to a significant reduction in malaria morbidity in the underfives^9–11^. Despite its effectiveness in the sub-Sahel region, SP-AQ-SMC cannot be used in East Africa since resistance against both drugs in the combination is common^13^. Instead an effective artemisinin-based combination therapy such as dihydroartemisinin-piperaquine (DP) can probably be used in the region^14–17^.

Studies show DP to be highly efficacious and well tolerated for treatment of uncomplicated malaria^14,18^. The fast-acting dihydroartemisinin rapidly clears the malaria parasites^19,20^, whereas the long-acting piperaquine with a terminal elimination half-life of 20–30 days, kills the residual parasites and also provides a long post-treatment prophylactic effect^16^. The long DP post-treatment prophylactic effect offers significant benefits over other ACTs^16,17^. In Burkina Faso and Uganda, DP-SMC provided excellent protection against malaria and was well tolerated^15,17,21^. Commonly reported DP adverse events (AEs) include gastrointestinal upset (nausea, vomiting, abdominal pain and diarrhea) as well as dizziness, headache and disturbed sleep.

Tanzania recently adopted SMC strategy using DP in a supplement 2018–2022 Malaria Control Strategic Plan to be used in areas with highly seasonal malaria transmission including Mtwara, and Ruvuma regions^22,23^. No study has however been conducted before to evaluate the protective effectiveness of the strategy in the country before it is scaled out, considering the fact that the targeted settings have extended seasonal malaria transmissions unlike the short seasonal transmissions in the Sahel region. This study therefore, assessed the safety and protective effectiveness of DP when used as SMC in underfive children.

## Results

### Baseline characteristics of the study participants

**Figure 1:**
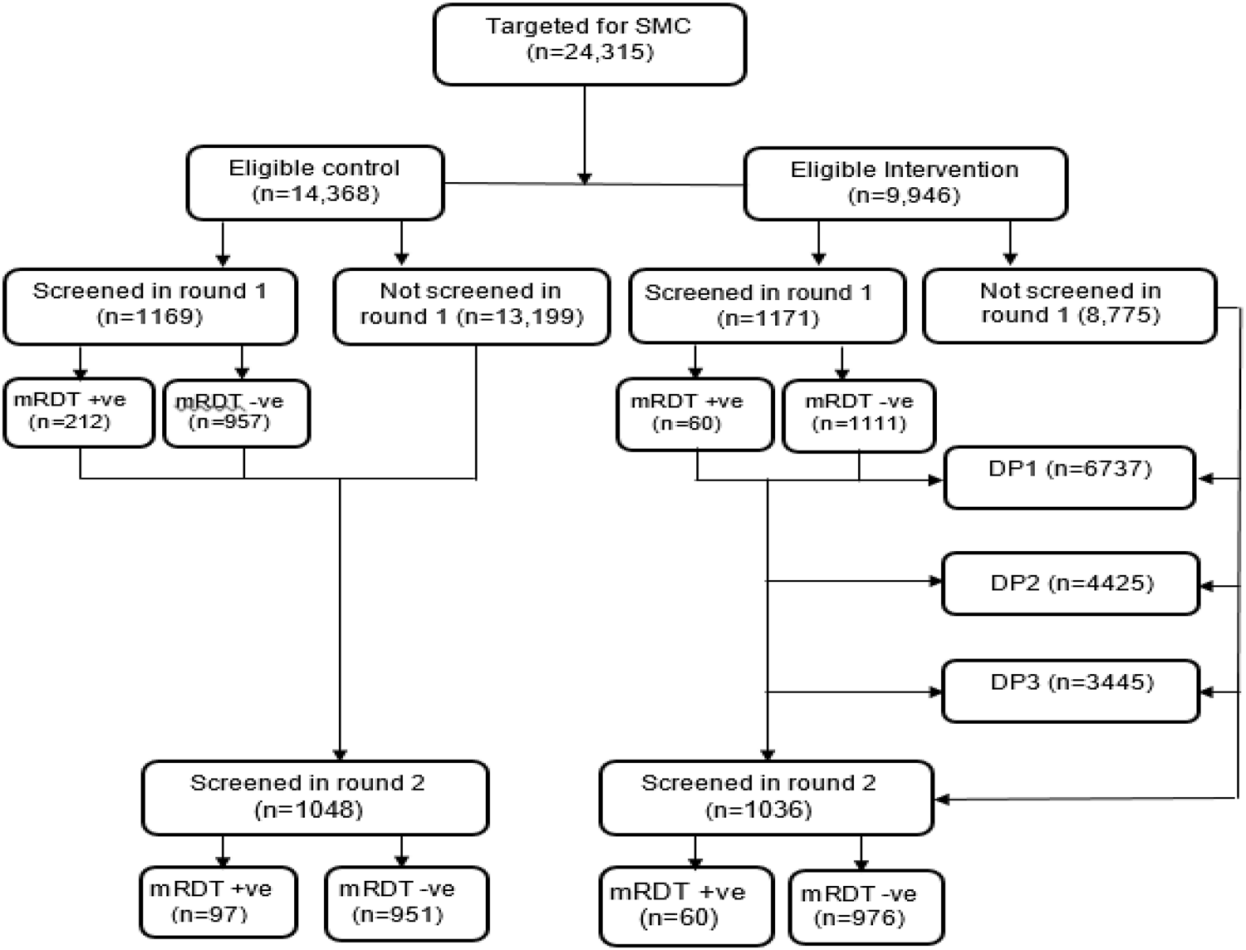
Trial profile showing study population screening for malaria using mRDT tests, test results (mRDT positive (+ve) and negative (−ve)) and population that received study drug (DP) at the three time points.

A total of 2340 participants with median (inter quartile range) age of 2.25 years (IQR: 1.20–3.43), 1215 (51.9) girls and 1635 (69.90%) from Masasi were screened for malaria parasites using both mRDT and microscopy. The participants’ baseline characteristics in both arms are presented in Table 1. The children in the intervention and control clusters had statistically significantly different median age, mean weight, mean height, mean body mass index (BMI), and mean BMI for age (BAZ). Bed net coverage was significantly higher in the intervention clusters. The trial profile is presented in Fig. 1.

**Table 1:** Characteristics of the participants in the intervention and control clusters at baseline.

Variable	Overall	Intervention clusters	Control clusters	*Test*, *p*-value
Age, median (IQR), years	2.25 (1.20–3.43)	2.34 (1.37–3.58)	2.14 (1.07–3.17)	*z* = − 3.806,* p* < 0.001
Sex, boys, n (%)	1125 (48.1)	559 (47.4)	569 (48.7)	0.381, *p* = 0.537
Girls, n (%)	1215 (51.9)	616 (52.6)	599 (51.3)	
Weight (kg), mean (SD)	11.50 (3.0)	11.68 (3.0)	11.33 (2.9)	*t* = − 2.795, *p* = 0.005
Height (cm), mean (SD)	81.95 (13.1)	83.10 (13.1)	80.87 (13.0)	*t* = − 4.048, *p* < 0.001
BMI, mean (SD)	17.20 (3.30)	17.03 (3.60)	17.37 (3.10)	*t* = 2.410, *p* = 0.016
BAZ, mean (SD)	0.63 (1.60)	0.51 (1.70)	0.74 (1.60)	*t* = 3.204, *p* = 0.001
WAZ, mean (SD)	− 0.639 (1.21)	− 0.68 (1.17)	− 0.59 (1.25)	*t* = 1.657, *p* = 0.098
HAZ, mean (SD)	− 1.678 (1.53)	− 1.73 (1.578)	− 1.62 (1.47)	*t* = 1.649, *p* = 0.099
Hb concentration, g/dL mean (SD)	10.9 (1.4)	11.0 (1.4)	10.9 (1.4)	*t* = − 1.795, *p* = 0.073
Bednets coverage, n (%)	1412 (84.7)	782 (93.1)	630 (76.3)	*Χ*^*2*^ = *91.2, p* < *0.001*
Socio-economic status: Low	421 (34.0)	223 (31.5)	198 (37.4)	*Χ*^*2*^_*(2)*_ = *4.850, p* = *0.088*
Moderate	410 (33.1)	241 (34.0)	169 (31.9)	
High	408 (32.9)	245 (34.6)	163 (30.7)	

Significant values are in italics.

### SMC delivery

A total of 14,607 DP doses were administered over the three months. Assuming the study population to be constant, the SMC coverage was 67.7% (6737/9946), 44.5% (4425/9946), and 34.6% (3445/9946) in the first, second, and third month of delivery, respectively. Table 2, present the proportions of the eligible participants who failed to take DP in the three rounds of SMC. Refusal to take the study drug was the major reason for failing to take DP by the majority of the participants in all the three rounds.

**Table 2:** Proportions of children who missed the DP in the three rounds of SMC and its related factors.

Variable	Round 1	Round 2	Round 3
Refused	2688 (83.76%)	5309 (96.16%)	6384 (98.20%)
Used AL recently	261 (8.13%)	147 (2.66%)	72 (1.11%)
Sickness	195 (6.10%)	48 (0.87%)	31 (0.48%)
Not around	47 (1.46%)	11 (0.19%)	8 (0.12%)
Below 3 months	13 (0.41%)	4 (0.07%)	6 (0.09%)
Used septrine	5 (0.16%)	2 (0.04%)	0 (0.0%)
Total	3209 (100.0%)	5521 (100.0%)	6501 (100.0%)

### Effectiveness of the SMC on malaria prevalence by type of intervention

**Figure 2:**
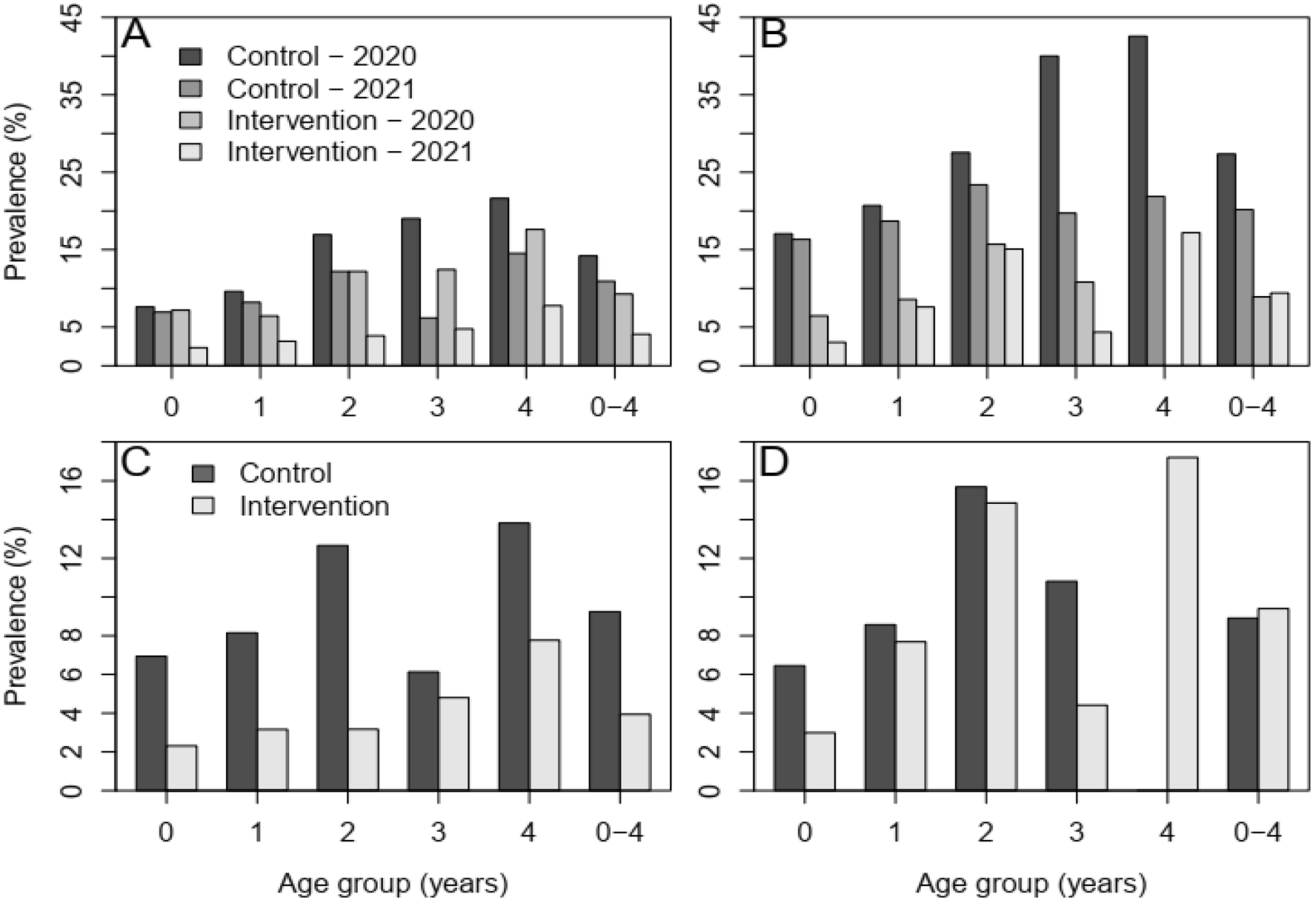
Distribution of mRDT positivity by age group. The top panels show the prevalence in the control and intervention groups before and after deployment of DP in Masasi (**A**) and Nanyumbu (**B**) Districts. Panels C (Masasi) and D (Nanyumbu) shows the prevalence of mRDT positive in 2021 by study group (control vs. intervention).

A total of 20 villages from 20 wards were selected for the evaluation of the impact of the intervention (10 controls and 10 interventions). Before the administration of DP-SMC, the prevalence of malaria by mRDT test was 13.75% (161/1171) in the intervention clusters and 18.14% (212/1169), in the control clusters, *p* = 0.004. Likewise, by microscopy the prevalence was 7.2% (84/1171) in the intervention clusters and 10.9% (128/1169) in the control clusters, *p* = 0.001. The within arms analysis showed that, malaria prevalence by mRDT declined to 5.79% (60/1036) in the intervention clusters following the administration of the three rounds of SMC, *p* = 0.003, and in the control clusters it declined to 9.26% (97/1048), *p* = 0.003. By microscopy the malaria prevalence declined significantly to 3.7% (38/1039) in the intervention clusters (*p* < 0.001), and to 3.8% (40/1045) in the control clusters, *p* < 0.001. But between arms analysis showed that, after the period of three months of SMC administration in the intervention clusters, the proportion of malaria decline was not statistically significantly different between the intervention and control clusters, (*p* = 0.838). Likewise, although the univariate analysis showed that the intervention clusters had significantly low prevalence of malaria than the control clusters (PR 0.42, *p* < 0.001), multivariate models which control for other factors indicated that malaria prevalence was 23% lower in the intervention than in the control arm, although the difference was not significant, *p* = 0.189, Table 3. Distribution of mRDT positivity before and after the three rounds of SMC in the intervention and control clusters by age group and District is presented in Fig. 2. Panels A and B show that the prevalence was higher in the baseline in both arms and decreases substantially in year 2 noticeably in the intervention arm in Masasi District. Furthermore, the analysis for period post intervention (Fig. 2C, D) show that, with exception of the 4 years age group, malaria prevalence in other age groups was significantly lower in the intervention than in the control clusters.

**Table 3:** Univariable and multivariable model showing prevalence ratios (PR) of mRDT different variables.

	Univariable			Multivariable		
Variable	PR	95%CI	*P*-value	PR	95%CI	*P*-value
Received—no intervention	1			1		
Intervention	0.42	0.32–0.55	< 0.001	0.77	0.53–1.13	0.189
Year-2020	1			1		
-2021	0.47	0.39–0.57	< 0.001	0.53	0.42–0.67	< 0.001
Randomized—control	1			1		
intervention	0.72	0.60–0.85	< 0.001	0.74	0.61–0.92	0.005
Age (years)	1.23	1.15–1.30	< 0.001	1.23	1.15–1.30	< 0.001
District—Masasi	1			1		
Nanyumbu	1.73	1.45–2.05	< 0.001	1.72	1.44–2.04	< 0.001

### Effectiveness of the SMC on incidences of uncomplicated and severe malaria

Malaria cases declined from 960 (95%CI 944–975) cases in 2020 to 692 (95%CI 679–706) per 1000 person years in the control arm, while in the intervention, the cases declined from 887 (95%CI 869–905) to 612 (95%CI 597–628) per 1000 person years during the same period. Compared to year 2020, malaria cases declined by 27.9% (95%CI 24.7–31.5) in the control arm, while in the intervention it declined by 31.0% (95%CI 27.4–34.9).

Results from a Poisson with wards fitted as random effect and data from year 2019 contributing to the baseline, showed that there was no significant difference between wards randomized to interventions compared to controls (*p* = 0.427). However, wards which were randomized and received DP intervention in 2021 had significantly lower incidence rate by 3.87% (95%CI 0.3–7.3), *p* = 0.034, Table 4. Furthermore, malaria incidence was decreasing across the years, it was higher in Nanyumbu District and during the quarters corresponding to rainy seasons particularly April–June when compared to January–March. The significant alpha showed that the random effect model was significantly different from the normal Poisson model.

**Table 4:** Factors associated with risk of malaria among patients who attended at the outpatient clinics in Nanyumbu and Masasi Districts between 2019 and 2021.

Variable	IRR	95% CI	*P*-value
Randomized to Control	1		
Intervention	1.201	0.764–1.898	0.427
Year 2019	1		
2020	0.871	0.856–0.886	< 0.001
2021	0.627	0.612–0.642	< 0.001
Received no DP	1		
Received DP	0.961	0.927–0.997	0.034
District—Masasi	1		
Nanyumbu	2.916	1.785–4.761	< 0.001
Quarter (January–March)	1		
April–June	1.402	1.377–1.427	< 0.001
July–September	0.736	0.720–0.751	< 0.001
October–December	0.272	0.264–0.280	< 0.001
Alpha	0.250	0.137–0.455	< 0.001

### Effectiveness of the SMC on prevalence of anemia

**Figure 3:**
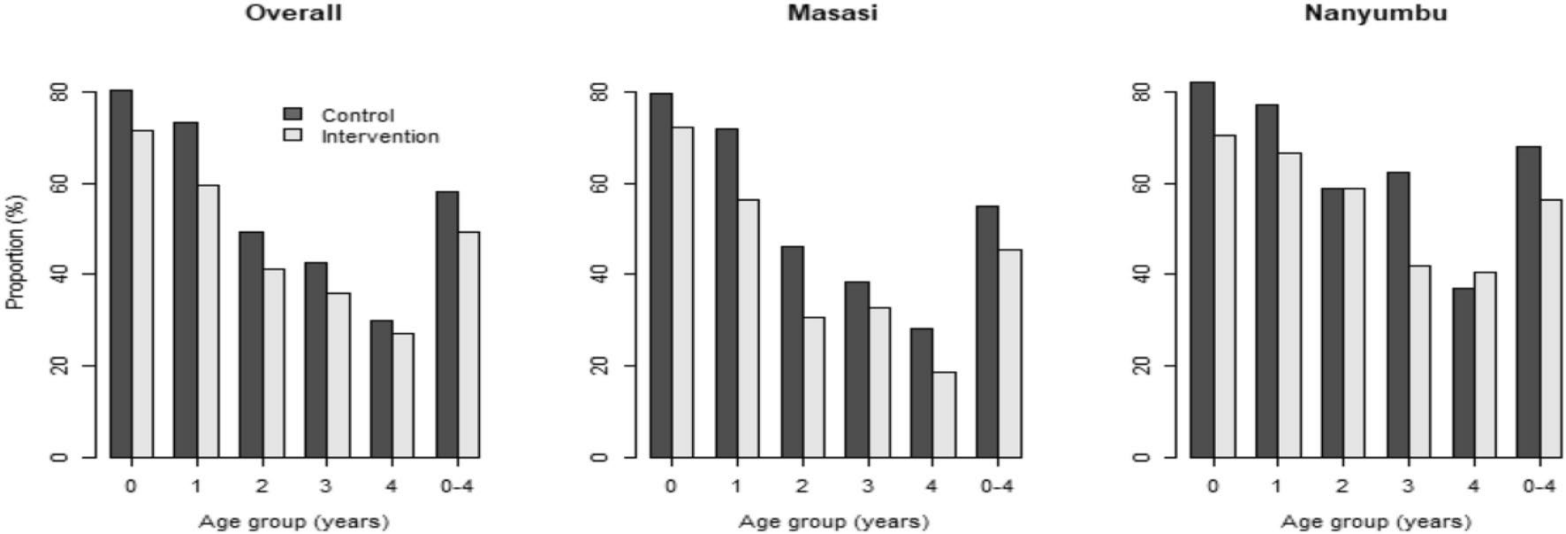
Overall and District specific distribution of anemia by age groups.

About 54.00% (1196/2219) of the participants had anemia before the administration of the SMC, and the prevalence was significantly different between the intervention (53.14% (558/1050)) and control clusters (54.62% (638/1168)), *x*^2^ = 0.49, *p* = 0.485. Following the DP administration, the prevalence of anemia declined to 49.18% (511/1039) in the intervention clusters, whereas in the control clusters it increased to 58.02% (608/1048), and the difference was significant (*x*^2^ = 16.4, *p* < 0.001). The prevalence of anemia after the SMC administration by age groups and Districts between control and intervention clusters is presented in Fig. 3. In all the age groups, except the age of 2 and 4 years in Nanyumbu District, the prevalence of anemia was significantly lower in the intervention than in the control clusters.

### Factors associated with hemoglobin concentration in the study population

Table 5 shows factors associated with hemoglobin (Hb) concentration in the study participants after the intervention. With all factors remaining constant, the mean Hb in the study area was 10.17 g/dL (95%CI 10.06–10.28), and this was significantly different from zero, *p* < 0.001. Children with positive mRDT tests had lower Hb by 0.80 g/dL when compared to those with negative tests. The mean Hb concentration was higher by 0.34 g/dL in clusters that received the intervention compared to the controls. On the other hand, an increase in age by one year was associated with an increase in Hb concentration by 0.38 g/dL. Furthermore, the Hb concentration in girls was higher by 0.22 g/dL compared to that of boys, whereas the Hb concentration in Nanyumbu was lower by 0.32 g/dL compared to that of Masasi District.

**Table 5:** Multivariable model showing factors associated with haemoglobin concentration among the study participants.

Variable	Coefficient	95%CI	*P*-value
Sex—Female	0.22	0.14–0.29	< 0.001
Age (year)	0.38	0.35–0.41	< 0.001
mRDT positive	− 0.80	− 0.92– − 0.68	< 0.001
Received—intervention	0.34	0.19–0.50	< 0.001
Year 2021	− 0.22	− 0.33– − 0.11	< 0.001
Randomized to intervention	− 0.01	− 0.12–0.10	0.818
District—Nanyumbu	− 0.32	− 0.40– − 0.24	< 0.001
Intercept	10.17	10.06–10.28	< 0.001

Table 6 shows the prevalence ratios for different variables associated with anaemia which are similar to those presented in Table 5. Clusters randomization at the baseline were shown not to have the impact on the prevalence ratio of cases versus controls (*p* = 0.430) as well as year of the survey (*p* = 0.071). Variables that were associated with decrease in prevalence ratio were sex (girls), age, and receiving interventional drug, while mRDT positive individuals and living in Nanyumbu District were associated with increased prevalence ratios, Table 6.

**Table 6:** Multivariable model showing prevalence ratios (PRs) of different variables associated with anaemia (Hb < 11 g/dl) in the study area.

Variable	PR	95%CI	*P*-value
Sex—Boys	1		
–Girls	0.88	0.81–0.95	0.002
Age (year)	0.78	0.76–0.81	< 0.001
mRDT—Negative	1		
–Positive	1.43	1.27–1.60	< 0.001
Received—No intervention	1		
–Intervention	0.81	0.69–0.96	0.015
Year—2020	1		
–2021	1.11	0.99–1.24	0.071
Randomized—control	1		
– Intervention	1.05	0.93–1.18	0.43
District—Masasi	1		
–Nanyumbu	1.22	1.12–1.33	< 0.001

### Safety of the SMC

A total of 60 (5.79%) participants had adverse events after the administration of the SMC. In total, 64 AEs were reported, Table 7. Vomiting, fever, and abdominal pain were the major reported AEs, and all were mild and self-limiting.

**Table 7:** Distribution of the adverse events after administration of SMC in Nanyumbu and Masasi Districts.

Variable	Overall, n (%)	Nanyumbu, n (%)	Masasi, n (%)
Vomiting	19 (29.59)	9 (25.71)	10 (34.48)
Fever	15 (23.44)	8 (22.86)	7 (24.14)
Abdominal pain	10 (15.63)	7 (20.00)	3 (10.34)
Slumbering	7 (10.94)	2 (5.71)	5 (17.24)
Headache	5 (7.81)	2 (5.71)	3 (10.34)
Rashes	5 (7.81)	4 (11.43)	1 (3.45)
Flue	1 (1.56)	1 (2.86)	0 (0)
Cough	1 (1.56)	1 (2.86)	0 (0)
Hallucination	1 (1.56)	1 (2.86)	0 (0)
**Total**	**64 (100)**	**35 (100)**	**29 (100)**

Significant values are in bold.

## Discussion

Tanzania recently adopted the SMC strategy for control of malaria in areas with an extended highly seasonal malaria transmission. Therefore, this operational study assessed the safety and protective effectiveness of the strategy using DP in Masasi and Nanyumbu Districts before the scale-out of the strategy. The findings showed that after the three rounds of DP-SMC, malaria prevalence declined significantly in the intervention clusters as shown in the univariate analysis (PR = 0.42, *p* < 0.001). It was also interesting to note the lower risk of 23% in the intervention when compared to the control, although it was not significant (*p* = 0.189). The lack of significance can be attributed to the low statistical power due to lower effect of the intervention as a result of lower compliance to SMC delivery in all the three rounds. The DP coverage in the first round was above 60%, however, it declined to 45% and 35% in the second and third rounds, respectively. The study was conducted during the COVID-19 pandemic. The first round of DP delivery was conducted in early March 2021 before the country had adopted COVID-19 vaccines as a measure to control the pandemic. The government of Tanzania officially approved the use of vaccines against COVID-19 on 31st March 2021, just before the delivery of the second and third rounds of DP, in April and May 2021. Due to population skepticism and misconceptions around the COVI-19 vaccines at the time, the study population perceived DP delivery to be used to also deliver COVID-19 vaccine, and therefore, refused to take the medicine. This negatively affected the SMC coverage and hence its effect.

Furthermore, when assessed by age group, in all the age groups except in the year 4 group in Nanyumbu District malaria prevalence was significantly lower in the intervention than in the control clusters (Fig. 2C, D). Similar findings have been reported in the Sahel countries^9–11,15^. However, when comparing malaria prevalence between the year 2020 and 2021 (before and after three rounds of DP), both in the control and intervention clusters the malaria prevalence was lower in the 2021 than 2020 in all the age groups and both Districts. Likewise, malaria incidences declined significantly both in the control and intervention clusters between 2020 and 2021. However, there was no significant difference in malaria incidences when compared between the control and intervention clusters in 2021 after the three rounds of DP in the intervention clusters. The corresponding decline of malaria prevalence and incidences in the control clusters similar to intervention clusters may be due to different factors especially weather driven factors which may have affected all of the study arms^24,25^. Clusters were randomized to ensure an evenly distribution of control and intervention clusters in the study Districts, with evaluation villages/sites been selected in such a way that there was buffer zones to prevent contamination from adjacent villages/wards with different intervention. Thus, probably climatic factors that influence mosquito bleeding and hence malaria transmission such as decreased rainfall and temperature change out of the optimal range may have contributed to the decline of malaria in both arms. On the other hand, low DP-SMC coverage in the intervention clusters may have probably led to the lack of significant difference in malaria incidences between the intervention and control clusters.

Anemia is one of the major complications of malaria infection^26–28^. At the baseline more than half of the study children had anemia, and was not statistically significantly different between arms. After the three rounds of SMC, the anemia prevalence declined significantly in the intervention clusters, and increased significantly in the control clusters. Analysis by age groups showed that with the exception of the age groups of 2 and 4 years in Nanyumbu District, anemia prevalence was significantly lower in the intervention than in the control clusters. Studies in other settings have also shown a significant decline of anemia prevalence after the SMC intervention^9–11^. However, in this study it is not clear why in the control clusters although the malaria prevalence declined significantly but the anemia prevalence increased. Nonetheless, in this study factors including having malaria infection and residing in Nanyumbu District were positively associated with anemia, whereas, sex especially female, increasing age, and having received the DP-SMC were negatively associated with anemia.

The DP-SMC was safe and well tolerated. Only 6.0% of the participants in the intervention clusters had adverse events. Vomiting, fever and abdominal pain were the adverse events reported most frequently. All the adverse events were mild and self-limiting. Similar findings have been observed in other studies^14,15^.

Despite its strength the study had limitations. The study did not quantify the effect of climatic factors on malaria transmission and prevalence during the study period. This would have aided in understanding the effect of SMC alone on malaria prevalence. Furthermore, low coverage/distribution of DP in all the three rounds might have contributed to low impact of DP observed in this study.

## Conclusion

The DP was safe and effective for using as SMC for control of malaria in Masasi and Nanyumbu Districts. The observed adverse events were mild and self-limiting. The drug can therefore, be used in settings of Tanzania with extended seasonal malaria transmission to reduce the burden of malaria.

## Materials and methods

### Study area

**Figure 4:**
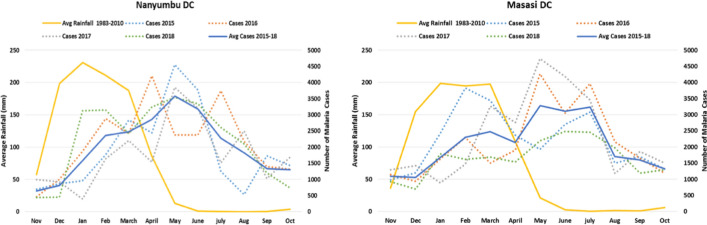
Weather characteristics of Nanyumbu and Masasi Districts.

The study was conducted in rural settings of Nanyumbu and Masasi Districts, Mtwara region. Nanyumbu District has 14 wards, and Masasi District has 22 wards. The Districts had a projected population of 82,740 children aged 3–59 months by 2019, 31,564 in Nanyumbu and 51,176 in Masasi District. There were 44,319 households in Nanyumbu by 2018, and Masasi had 73,998. Nanyumbu has 19 health facilities and Masasi has 40. The Districts have an average annual rainfall of 939 mm and a temperature of 25.4 °C. The rainfall occurs between January and April, and accounts for more than 60% of the average annual rainfall. Malaria transmission is highly seasonal with > 60% of infections occurring between March and July (Fig. 4). A total of 36 clusters, 18 interventional and 18 control were used. A buffer was set between evaluation sites to minimize contamination from surrounding wards/villages that were receiving different intervention.

### Study design

It was an open cluster randomized study with two arms (intervention and control). Clusters were randomized using Research Randomizer version 4 computer software (Wesleyan University, Connecticut, USA)^29^, with a ward used as a randomization unit. Eligible children in the intervention clusters received DP monthly for three consecutive months (three rounds) regardless of their malaria infection status, whereas those in the control clusters received artemether-lumefantrine upon having a microscopy/malaria rapid diagnostic test (mRDT)-confirmed malaria. A health facility and villages near the centre of each of the study wards was selected as an evaluation point within the ward. This selection was done to minimize contaminations (buffer zone) from surrounding wards/villages receiving different intervention. Community health workers (CHWs) administered DP door-to-door. A round of DP administration was completed within 3–5 days. Safety of DP was assessed at the end of each SMC round. Seven weeks after the third round of SMC a cross-sectional survey was carried out to assess the strategy’s effect on malaria morbidity.

### Study population

Underfive children were involved. The inclusion criteria were being a permanent resident of the catchment area, aged 3–59 months, willingness of the caregivers to allow their children to participate in the study, and ability to swallow oral medications. Participants were excluded if had an acute febrile illness or severe illness that impairs ability to take oral medication; were receiving cotrimoxazole (septrine) prophylaxis; had received a dose of antimalarial drugs within the past month; and had a history of allergy to DP.

### Procedures

#### Assessment of baseline social characteristics and malariometric surveys

Initially, the wards sampled for the study were visited. The health facilities near the centre of the wards were identified and the populations they serve were estimated. Likewise, a study village(s) within which the facility is located was identified. Thereafter, a social mobilization campaign was conducted through local meetings and announcements in the houses of worship to explain the study start date, procedures, its significance, and the importance of community participation. Thereafter, a demographic and malariometric survey was conducted in July 2020 to determine the socio-economic characteristics of the households, and the baseline malaria morbidity including the malaria prevalence and anemia.

#### SMC delivery

The SMC administration began in March and ended in June 2021. But before the SMC delivery, the CHWs were trained on the study procedures including taking child’s history such as any intake of antimalarial drugs in the preceding month, child’s age, checking for fever, selecting a correct dose, administering the first-dose of DP, and instructing caregivers how to administer the subsequent doses.

DP (D-ARTEPP, Guilin Pharmaceutical, Shanghai Co., Ltd., China) was administered based on the age as follows: 3–6 months, 1 (20 mg/160 mg) tablet; 7–24 months, 1^1^/_2_(20 mg/160 mg) tablets; and 25–60 months, 1 (40 mg/320 mg) tablet once a day for 3 days. For children who could not swallow a whole tablet dispersible tablets were dissolved in water and administered. After taking DP the participants were observed for 30 min, and if vomiting occurred within this period the dose was re-taken. The first-dose was administered by CHWs and the caregivers administered the subsequent doses.

#### DP-SMC safety assessment

The adverse events (AEs) were assessed by directly questioning the caregivers. The questions inquired on any symptoms and signs that emerged following the DP administration. When clinically indicated, patients were evaluated and treated for the emerging condition appropriately. An AE was defined as any unfavorable, unintended sign, symptom, syndrome or disease that developed or worsened with the use of a medicinal product, regardless of whether it was related to the medicinal product. A serious adverse event was defined as any untoward medical occurrence that at any dose: (1) resulted in death, was life threatening; (2) required hospitalization or prolongation of hospitalization; (3) resulted in a persistent or significant disability or incapacity; or (4) a congenital anomaly or birth defect.

#### Assessment of SMC effect on malaria morbidity

The cross-sectional survey was carried out in both the intervention and control clusters. Samples of children for the survey were drawn from households falling within the centre of the cluster to minimize contamination from adjacent clusters. Screening for malaria morbidity was carried out at the satellite health facilities. Children’s height and weight were recorded. Finger prick blood samples were collected and used to assess the presence of malaria infection using mRDT, and thick and thin films for microscopy, and measurement of hemoglobin concentration. Axillary temperature was also taken.

### Laboratory analysis

Absolute methanol was used to fix thin smears. Thin and thick smears were air-dried, stained using 3% giemsa for 1 h, and examined under the microscopy at 100 × high power fields under immersion oil. Parasitemia was determined by counting the number of parasites present per 200 white blood cells (WBC) on a thick smear, and thereafter multiplied by 40 assuming a WBC count of 8000 per milliliter of blood. Slides were read independently by two technicians. A third reading was requested in case of a mismatch (positive versus negative or a difference in parasite density greater than 30%). A 10% of the slides was read by an independent microscopist for quality control.

Haemoglobin concentration was measured using a HemoCue Hb 201 + (HemoCue AB, Ängelholm Sweden), with a precision of +/− 0.3 g/dL. The HemoCue was calibrated every morning using a control cuvette at 16.0 ± 0.3 g/dL. Anaemia was classified as haemoglobin level < 11 g/dL (mild), < 7 g/dL (moderate) and < 5 g/dL (severe).

### Study outcomes

The outcomes were compared between the arms. The primary outcome was the prevalence of clinical malaria defined as the presence of fever (axillary temperature 3 37.5 °C) or a history of fever in the past 24 h and the presence of *P. falciparum* asexual parasitaemia at any density. Secondary outcomes included: (i) prevalence of malaria infection defined as the presence of asexual parasitaemia; (ii) prevalence of cases of uncomplicated and severe malaria reported at the health facilities; (iii) prevalence of anemia and; (iv) safety and tolerability measured by the occurrence of non-serious and serious AEs.

### Ethics

The Declaration of Helsinki and other regulations in Tanzania were observed throughout the conduct of the study. The ethics committee of the Muhimbili University of Health and Allied Sciences approved the study protocol. The communities and local authorities approvals were sought before the start of the study. Informed consent was sought from the caregivers of the participating children. The study is registered at the ClinicalTrial.gov with number NCT05874869.

### Sample size and power calculation

There is variation in malaria incidences between the two Districts, with the three years (2016–2018) average being 309 cases per 1000 in Nanyumbu and 222.5 cases per 1000 in Masasi Districts, respectively. In this study the assumption was that malaria incidences as confirmed by mRDTs would decrease by 40% in the interventional clusters. Assuming a correlation coefficient, *k* = 0.2, a power of 80% and an alpha (type one error) of 0.05, then a total of fourteen (14) clusters, seven (7) per arm would be required with a sample size of 106 participants per ward in Masasi, whereas for Nanyumbu a total of twelve (12) clusters, six (6) per arm with a sample size of 106 participants per ward was required. A 20% attrition rate was considered that resulted into a sample of 128 participants per cluster.

The 2017 malaria indictor survey indicated that malaria prevalence in Mtwara region as confirmed by mRDT in children aged 6–59 months was 14.8%^30^. To be able to detect the difference in the prevalence of malaria infection by 50% between the intervention and control areas, a sample size of 130 children from each of the clusters from the two arms was required to be sampled during the cross-sectional surveys. A 10% was considered as an attrition rate in each of the evaluation areas. These clusters were nested in clusters selected for incidence study.

### Data management plan

The data were collected using electronic case report forms in Redcap of Open data kit by tablet computers. Data were uploaded on a platform on daily basis. Spatial data (point coordinates for households and villages and other features from the study areas) were collected during the household visits using Global Positioning System inbuilt in tablet computers. Back-ups of data was made on a daily basis onto external hard disks and stored in a secured place separately from the building hosting data management section. Source documents (in case of paper work) were achieved.

### Data analysis

Data analysis was performed using Stata and R statistical packages. Exploratory methods (tabulations and plots) were used to explore the distribution and different patterns that were important in the selection of possible models and/or any transformation that would be required.

Different statistical models were used in modeling relationship between the response variables by the intervention groups and other explanatory variables. Estimates were presented together with 95% confidence intervals with a* p*-value < 0.05 considered significant.

Proportions were compared using *chi*-square test while logistic regression model was used to assess associations between binary outcome variable and explanatory variables. For the cross-sectional surveys data, survey analysis methods, which take into considerations potential clustering of data, were employed.

Malaria incidence cases collected through health facility system were used to determine the number of malaria events per ward catchment population size and presented per 1000-person years. All health facilities within the study ward were used to generate the monthly number of malaria cases, while the ward population size was obtained from the 2022 National Census survey. An annual growth rate of 1.2% and proportion of children below 5 years (12.3%) for Mtwara region derived from the 2022 National census survey were used to project the population size for years 2019–2021. For year 2021, health facility data was available for January–November, and a scale-up of population by 11/12 was used to account for cases for December 2021. Since only 2.3% records of malaria results were confirmed by BS or BS and mRDTs, we only used mRDT test results which were available for over 97% of the records. Malaria cases were then modeled as count data using Poisson model with ward as a random effect variable, a model which was determined to be superior to Poisson model by log-likelihood ration test. The estimated population of children aged 0–4 years from the study wards included in the model as an exposure variable during the modeling.

### Ethics approval and consent to participate

The approval to conduct this study was obtained from the Ethics Committee of the Muhimbili University of Health and Allied Sciences with approval number: MUHAS-REC-10-2019-062. Mtwara region, and Masasi and Nanyumbu District authorities provided the permission to conduct the study at the sites. Meetings were held with community, administrative, and religious leaders to explain the study motives and activities, and also sought community approval. Written informed consent was sought from the heads of the households.

### Supplementary Information

Supplementary Information.

## Data Availability

All relevant data are within the manuscript and its supporting information files.

## Abbreviations

ACTs: Artemisinin-based combination therapies
AEs: Adverse events
AL: Artemether-lumefantrine
AQ: Amodiaquine
BMI: Body mass index
BAZ: Body mass index for age
CHWs: Community health workers
CI: Confidence interval
DP: Dihydroartemisinin-piperaquine
HAZ: Height for age
Hb: Hemoglobin
HIV: Human immunodeficiency virus
mRDT: Malaria rapid diagnostic test
MUHAS: Muhimbili University of Health and Allied Sciences
PR: Prevalence ratio
SD: Standard deviation
SMC: Seasonal malaria chemoprevention
SP: Sulphadoxine-pyrimethamine
SSA: Sub-Saharan Africa
WAZ: Weight for age
WBC: White blood cells
WHO: World Health Organization
USA: United States of America
